# Simultaneous Quantitation of a Novel α_1_/β_1_-Blocker TJ0711 and Its Two Metabolites in Dog Plasma Using LC-MS/MS and Its Application to a Pharmacokinetic Study after Intravenous Infusion

**DOI:** 10.3390/pharmaceutics11010038

**Published:** 2019-01-18

**Authors:** Wenwen Zhu, Wanyu Liu, Haojv Li, Guojia Xu, Qian Li, Jiangeng Huang, Gao Li, Luqin Si

**Affiliations:** School of Pharmacy, Tongji Medical College, Huazhong University of Science and Technology, Wuhan 430030, China; wenwenzhu0116@hust.edu.cn (W.Z.); m201875230@hust.edu.cn (W.L.); lihaojv18@mails.ucas.ac.cn (H.L.); shzxgj11@gmail.com (G.X.); m201675278@hust.edu.cn (Q.L.); jiangenghuang@hust.edu.cn (J.H.); gao_li@hust.edu.cn (G.L.)

**Keywords:** TJ0711, LC-MS/MS, pharmacokinetics, metabolite, beagle dog

## Abstract

TJ0711∙HCl, which is a novel α_1_/β_1_ adrenoceptor blocking agent with a ratio of 1:1 for α_1_/β_1_, is designed to treat and prevent perioperative hypertension. M1 and M3 were identified as important metabolites in vitro for either antihypertension activity or the major metabolite production. In order to obtain a pharmacokinetic profile of both TJ0711 and its metabolites, a rapid, selective, and reliable LC-MS/MS method was developed and validated for simultaneous determination of TJ0711 and two metabolites in beagle dog plasma via efficiently separating two interferential metabolites M16 and M4 from M1 and M3, respectively. Chromatographic separation was achieved on a Waters CORTECS C18^+^ column (2.1 × 100 mm, 2.7 μm). The mass spectrometric detection was carried out in positive ion MRM mode with ESI^+^ source. Protein precipitation was used in sample preparation and provided good recovery without a matrix effect. Good linearity was observed at the ranges of 0.5–100 ng/mL for TJ0711 and M3, 0.1–20 ng/mL for M1. Additional validation results were within the acceptance limits followed U.S. FDA guidelines for bioanalytical method validation. This method was successfully applied to an intravenous infusion pharmacokinetic study of TJ0711 at dosing rates of 3, 6, and 12 µg/kg/min in anesthetized beagle dogs for the first time. TJ0711 and its two metabolites exhibited effective proportionality in the dosage of 3 to 12 µg/kg/min. Neither TJ0711 nor its metabolites showed significant differences in pharmacokinetic parameters such as *t*_1/2_, *CL*, and *V_ss_* among three dose groups.

## 1. Introduction

Hypertension has become an important risk factor for cardiovascular disease and the leading causes of death [1,2,3,4]. Perioperative hypertension is a common problem that 25% of hypertensive patients undergoing surgery might encounter [5]. Except for hypertensive patients, non-hypertensive patients are likely to develop a rise in blood pressure and tachycardia during induced anesthesia as well [6]. Clinically, perioperative hypertension is treated with four categories of hypotensive drugs including β blockers, angiotensin-converting enzymes (ACE) inhibitors, calcium channel blockers (CCB), and vasodilators [5,7,8]. For example, labetalol, which is a nonselective β blocker with α_1_ to β blocking ratio of 7:1, is used to reduce blood pressure while maintaining total peripheral blood flow, heart rate, and cardiac output [9]. Labetalol has a quick hypotensive effect within 2 to 5 min after intravenous administration [10]. However, the half-life of labetalol was approximately 5.5 h, which leads to the difficulty to titrate as continuous infusion [5]. Nicardipine is a CCB with rapid action [11,12]. Due to its augmentation of coronary blood flow and increasing cardiac output (CO), nicardipine can reduce cardiac and cerebral ischemia, balance myocardial oxygen demand, and supply after intravenous administration [13]. Nevertheless, caution should be taken during clinical use of nicardipine in the patients with hypohepatia, anoxemia, and intracranial hypertension since this drug is light-unstable and precipitates crystal in a high pH environment [8]. In addition to labetalol and nicardipine, other active agents such as esmolol (β blocker), clevidipine (CCB), and fenoldopam (vasodilator), are also used for perioperative hypertension in which each has advantages and disadvantages [5,8]. The ideal agent for treatment of perioperative hypertension should be rapid acting, predictably and easily titrated, safe, and inexpensive [6]. Therefore, it is of great clinical significance to develop a novel, safe hypotensive drug with high efficacy and minimal toxicity. 

**Figure 1:**
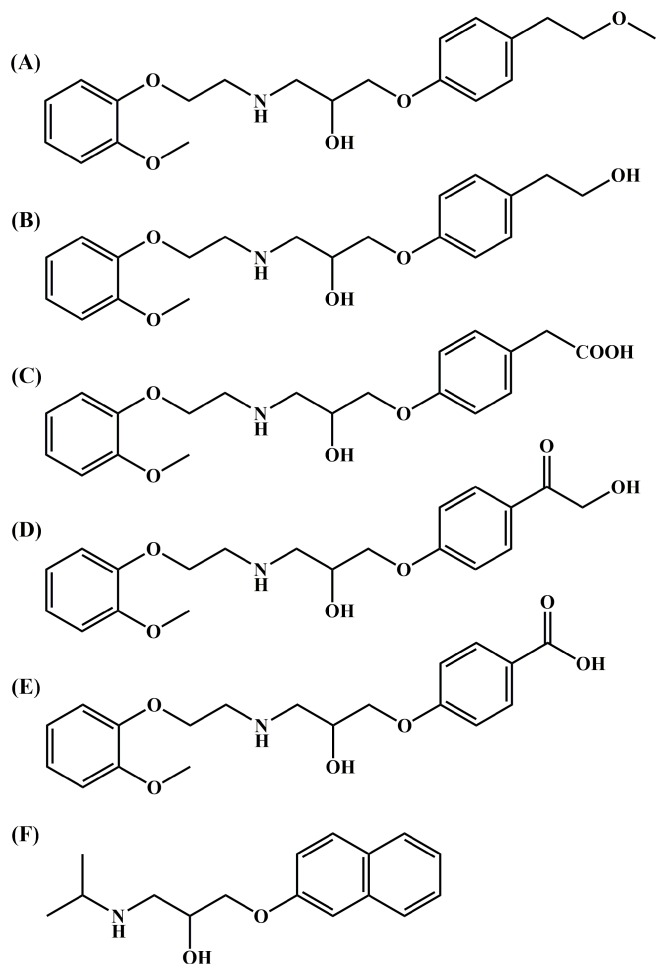
Chemical structures of (**A**) TJ0711, (**B**) M1, (**C**) M3, (**D**) M4, (**E**) M16, and (**F**) Propranolol.

TJ0711∙HCl (1-[4-(2-methoxyethyl)phenoxy]-3-[2-(2-methoxyphenoxy)ethylamino]-2-propanol hydrochloride), a novel α_1_/β_1_ adrenoceptor blocking agent with a ratio of 1:1 for α_1_/β_1_, is now under preclinical development (structure shown in Figure 1) [14,15,16,17,18]. As reported in previous research studies, TJ0711 could reduce diastolic blood pressure and systolic blood pressure in both spontaneously hypertensive rats and ouabain induced hypertensive rats following intravenous administration [19,20]. TJ0711 showed maximum hypotensive effects at 2 to 15 min and maintained for 25 to 75 min [20]. TJ0711 showed higher potency for reno-protection due to its adjunctive α_1_-blocking activities [20,21]. In the early pharmacokinetic studies, TJ0711 showed short half-lives of around 25 min in rats [22] and 45 min in dogs [15]. Due to the previously mentioned advantages, there is great potential to develop TJ0711 as a novel intravenous agent for treating perioperative hypertension.

In in vitro metabolism studies, a total of 33 metabolites were found including 19 phase I and 14 phase II metabolites [14,23]. Moreover, CYP2D6 was proposed as the major enzyme for generation of both metabolites (data unpublished). The criteria for selecting the compounds in pharmacokinetic study include: (1) pharmacologically active metabolites of TJ0711, (2) inactive metabolites that account for the majority of systemic exposure, and (3) metabolites that are associated with toxicity issues. After 120 min incubation with liver microsomes, both M1 and M3 accounted for more than 10% of the relative amounts, demonstrating that M1 and M3 were two primary metabolites in liver microsomes of animal species and humans [14]. Interestingly, it was also found that M1 exhibited nearly the same hypotensive effect compared to the parent while M3 showed slight pharmacological effect, which indicates M1 was an active metabolite (data unpublished). However, a large number of TJ0711 metabolites are not available and further mass balance study could be helpful for guiding major metabolite synthesis. As such, in the current work, pharmacokinetic study of these three analytes of interest was carried out. 

Several precious analytical methods have been reported for TJ0711 by our group using high-performance liquid chromatography (HPLC)-UV [24] and HPLC-fluorescence [22,25]. Lately, an LC-MS/MS method was established for the determination of TJ0711 after intravenous administration in conscious beagle dog plasma [15]. However, this method only quantitatively analyzed the concentration of TJ0711. In addition, previous research reported that inhalational anesthetics might affect the elimination of perioperative drugs [26]. It is also essential to investigate the pharmacokinetic profiles of TJ0711 under anesthesia situation. In this study, anesthetized beagle dogs were administered by intravenous infusion of TJ0711 to simulate the clinical drug administration during peri-operation. In the present study, a sensitive, selective, and rapid LC-MS/MS method was developed and validated for simultaneous quantification of TJ0711 and its two important metabolites (M1 and M3) in dog plasma. It was also the first time to investigate the pharmacokinetic profiles of TJ0711 and two metabolites in anesthetized beagle dogs following constant intravenous infusion of three doses of TJ0711.

## 2. Materials and Methods 

### 2.1. Chemicals and Reagents

TJ0711 hydrochloride, M1 hydrochloride, and M3 were synthesized in our laboratory. The purity of TJ0711, M1, and M3 was >99.8%, >97.3%, and >99.5%, respectively, which was measured by a high-performance liquid chromatography (HPLC) system with ultraviolet (UV) detector. Propranolol hydrochloride (internal standard, IS) was obtained from the National Institutes for Food and Drug Control (NIFDC, Beijing, China). The chemical structures of TJ0711, two metabolite M1, M3, and IS are shown in Figure 1. HPLC-grade acetonitrile (ACN) was purchased from Fisher Scientific (Fair Lawn, NJ, USA). Deionized water was freshly prepared by a Milli-Q purification system (Millipore, Billerica, MA, USA). All other chemicals and solvents were of an analytical grade and were obtained from commercial sources. The blank blood was collected from six beagle dogs via cephalic vein into EDTA dipotassium dihydrate (EDTA-K_2_) tubes (BD Biosciences, Franklin Lakes, NJ, USA) and centrifuged at 1200× *g* for 10 min at 4 °C to obtain blank plasma. The blank plasma was then stored at −80 °C until used.

### 2.2. Instruments and Analytical Conditions

A Shimadzu Prominence UFLC system (Shimadzu Corporation, Kyoto, Japan) coupled with an API 4000 QTrap^®^ triple quadrupole mass spectrometer (AB Sciex, Foster City, CA, USA) equipped with an electrospray ionization (ESI) source was used throughout the study. Chromatographic separation was achieved on a Waters CORTECS C18^+^ column (2.1 × 100 mm, 2.7 μm, Waters Co., Milford, MA, USA) equipped with a guard column (Security-Guard, C18^+^, 2.1 × 5 mm, 2.7 μm, Waters Co., Milford, MA, USA). The column temperature was maintained at 40 °C. The mobile phase consisted of 0.1% (v/v) formic acid in water (Solvent A) and ACN (Solvent B). The flow rate was maintained at 0.5 mL/min. The following gradient conditions were used: 0–2 min, 15–20% B, 2–4 min, 20% B, 4–5 min, 80% B, 5–6 min, 15% B. The injection volume was 10 μL and the auto sampler temperature was set at 4 °C. 

Mass spectrometric detection was performed in a positive electrospray ionization (ESI) mode. Quantification was conducted using a multiple reaction monitoring (MRM) mode and the following MRM transitions 376.2→252.2, 362.0→238.0, and 260.1→116.1 were used for TJ0711 (M3 yielded the same precursor and product ions as TJ0711), M1 and IS, respectively, with a dwell time of 150 ms for each MRM transition. The mass spectrometric parameters including de-clustering potential (DP), collision energy (CE), entrance potential (EP), and cell exit potential (CXP) for each compound were optimized as follows: DP: 91 V, CE: 29 V, EP: 10 V, CXP: 6 V for both TJ0711 and M3; DP: 91 V, CE: 37 V, EP: 10 V, CXP: 8 V for M1; DP: 62 V, CE: 25 V, EP: 10 V, CXP: 8 V for IS. The source parameters were optimized as follows: ion spray voltage, 5500 V, source temperature, 600 °C, curtain gas, 25 psi, nebulizer gas, 40 psi, auxiliary gas, 50 psi, collision activated dissociation gas, high, interface heater, on. Analyst 1.6.1 software (Applied Biosystem/MDS Sciex, Foster City, CA, USA) was used for the instrument control, data acquisition, and processing.

### 2.3. Preparation of Calibration Standards, Quality Control Samples

TJ0711, M1, M3, and IS stock solutions were prepared using 50% ACN (v/v). The concentrations of TJ0711, M1, M3, and IS were corrected by the purity and salt correction factor of each compound. The corrected concentration was 10 mg/mL for TJ0711 and 5 mg/mL for the others. Calibration curve and quality control working solutions were prepared by serially diluting stock solutions separately in 50% ACN, all of which contained appropriate concentrations of each analyte. Furthermore, 20 μL working solution was spiked with 480 μL blank dog plasma to obtain the required final concentrations of the calibration standard (CS) and quality control (QC) samples. The final concentrations of all analytes of interest in CS and QC samples are listed in Section 3.2.3 and Section 3.2.4. Stock solutions and working solutions were stored at 4 °C.

### 2.4. Sample Preparation

All plasma samples including CS samples, QC samples, and plasma samples from pharmacokinetic study were prepared by protein precipitation. In addition, 100 μL plasma was spiked into 500 μL ACN, which contained 20 ng/mL IS. After vortex for 5 min and centrifugation at 16,000× *g* for 10 min at 4 °C, 480 μL of the supernatant was aspirated and evaporated using an SPD1010 SpeedVac Concentrator (Thermo Fisher Scientific, Waltham, MA, USA) at 45 °C, 5.1 Vac for 2.5 h. The residue was reconstituted in 80 μL of 15% ACN in water.

### 2.5. Method Validation

The method validation was followed by the United States Food and Drug Administration guidance for bioanalytical method validation [27]. Selectivity, the limit of detection (LOD), lower limit of quantification (LLOQ), linearity of the calibration curve, accuracy, and precision, matrix effect, carry-over, dilution integrity, and stability were evaluated. Six-individual dog blank EDTA-K_2_ plasma samples (3 male and 3 female) were processed in order to investigate the specificity and selectivity of this method. The peak area of interference should be ˂20% of the area of analytes in LLOQ samples and ≤5% for IS. LOD was defined as the concentration of which the signal to noise ratio (S/N) was larger than 3. LLOQ was defined as the lowest concentration of the calibration curve that yielded a peak with an S/N ≥ 10 while the accuracy should be 80% to 120% and precision should not exceed 20%. The linearity of the calibration curves was evaluated using eight calibration standards for each analyte in three different batches on three separate days. The calibration curves were plotted using the ratio of each analyte peak area over the internal standard peak area versus the nominal concentration using a weighting factor of 1/x^2^. To assess intra-assay and inter-assay accuracy and precision, LLOQ and three concentration QC level (low, medium, and high) samples were prepared along with the calibration curves on three separate days. According to the guidelines of the FDA, the error of accuracy and coefficient of variation (CV) should not exceed 15% for all CS and QC samples, except for the LLOQ (<20%). The matrix effects were evaluated by comparing the peak area of analytes of QC samples prepared from six-individual dog blank plasma with a peak area of neat solutions. Relative recovery was assessed by calculating the analyte peak area in spiked samples before extraction over the analyte peak area in spiked samples after extraction. Carry-over was investigated by running a double blank sample after the upper limit of quantitation (ULOQ) sample. The dilution integrity was evaluated by diluting dog plasma at the concentration 5-fold or 10-fold HQC with blank plasma using a ratio of 1:4 or 1:9, respectively. In this study, the stability of analytes was evaluated in both neat solutions and biological specimens. The stock solutions and working solutions were determined after 24 h storage in room temperature or 34 days storage in 4 °C. Besides, the stability of analytes in dog plasma was investigated by determining the concentrations of LQC and HQC samples after they had been kept at room temperature for 24 h, at −80 °C for 30 days, or after three freeze/thaw cycles. The IS was spiked after the storage for stability determination. The stability of TJ0711 and its metabolites in the processed samples was evaluated at auto sampler temperature of 4 °C for 24 h. For this situation, the IS has been added with a precipitation reagent for sample clean-up. The analytes were considered to be stable in biological specimens if the concentrations determined were 85% to 115% of the original concentration. The stability of analytes in whole blood was assessed as well.

### 2.6. Application of the Method to Pharmacokinetics of TJ0711 in Beagle Dogs

The validated method was applied to the pharmacokinetic study of TJ0711 in beagle dogs. The Animal Ethics Committee in Tongji Medical College, Huazhong University of Science and Technology (Wuhan, China) approved this study (Ref No. 00221434). Six beagle dogs (3 male and 3 female, license number: SYXK (Hubei Province)-2016-0057) weighing 10 ± 2 kg were anesthetized using isoflurane (RWD Life Science, Shenzhen, China) with Animal Anesthesia Ventilator (MATRX VIP3000, Midmark Animal Health, Versailles, OH, USA). Thereafter, TJ0711 hydrochloride, prepared in normal saline, was administrated by intravenous infusion at a dose rate of 3 μg/kg/min. After every 10 day washout period, another dose (6 or 12 μg/kg/min) was administrated. Approximately 1 mL blood samples were withdrawn via the cephalic vein at 0, 10, 20, 40, 60, 90, 120, 180, 240, 260, 280, 300, 360, 420, 480, and 600 min for 3 and 6 µg/kg/min and at 0, 10, 20, 40, 60, 90, 120, 180, 240, 260, 280, 300, 360, 480, 600, and 720 min for 12 μg/kg/min after dosing. Blood samples were collected in EDTA-K_2_ tubes and centrifuged at 1200× *g* for 10 min at 4 °C. The plasma was isolated into EP tubes and stored at −80 °C until analyzed.

### 2.7. Pharmacokinetic Analysis

The pharmacokinetic parameters were estimated using a non-compartmental model with Phoenix WinNonlin 6.4 software (Pharsight Corporation, Princeton, NC, USA). Maximum plasma concentration (*C_max_*) and time to reach *C_max_* (*T_max_*) were obtained directly from plasma concentration data. The area under the concentration versus time curve (including *AUC*_0–*t*_ and *AUC*_0–∞_), half-life (*t*_1/2_), steady-state volume of distribution (*V_ss_*), and clearance (*CL*) were calculated by WinNonlin 6.4 software. The lambda z ranges were calculated by WinNonlin software automatically and 3 to 6 points were used for half life calculation in individual dogs. Linear trapezoidal method was used for calculation of *AUC*. Then a power function model was applied to the pharmacokinetic parameters (*C_max_* and *AUC*_0–∞_) over the dose range by MATLAB R2017a software (MathWorks Inc., Natick, MA, USA) to assess the dose proportionality. The power function model was presented as PK = α × (Dose)^β^. It is assumed that the natural ln-transformed pharmacokinetic parameters are linearly related to natural ln-transformed dose: ln(PK) = α + β × ln(Dose) [28]. The slope of the line represents constant β, which was used for examining the proportionality and 95% confidence interval (CI) for β was calculated. One-way ANOVA followed by the LSD post hoc test was carried out for PK parameters as a comparison using SPSS Version 13.0 software (SPSS, Chicago, IL, USA).

## 3. Results and Discussion

### 3.1. Method Development

In our study, chromatographic conditions, mass spectrometric (MS) procedures, and a sample pretreatment method were optimized to obtain symmetrical peak shape, maximize the MS response, and achieve more accurate quantification. The chromatographic conditions including organic phase, additives, and gradient profiles were investigated. ACN was chosen because of the better chromatographic separation and improved peak shape compared to methanol. In previous LC-MS/MS method, isocratic elution was applied using ammonium formate solution as aqueous phase [15]. Unfortunately, ammonium formate failed to provide satisfying sensitivity and good peak shape for M1 at LLOQ on the Waters CORTECS C18^+^ column. Therefore, 0.1% formic acid was chosen as an additive in aqueous phase due to higher sensitivity and lower background interference. It was worth noting that, except M3, there was another metabolite (M4), which yielded the same precursor and product ions as the parent, while M16 yielded the same precursor and product ions as M1 [14]. Gradient conditions were developed to efficiently separate these two metabolites from TJ0711, M1, and M3 with less matrix effect, shorter analysis time, and better chromatographic separation. In addition, different concentrations of formic acid had little effect on the MS response and peak shape of three analytes under the developed gradient conditions.

**Figure 2:**
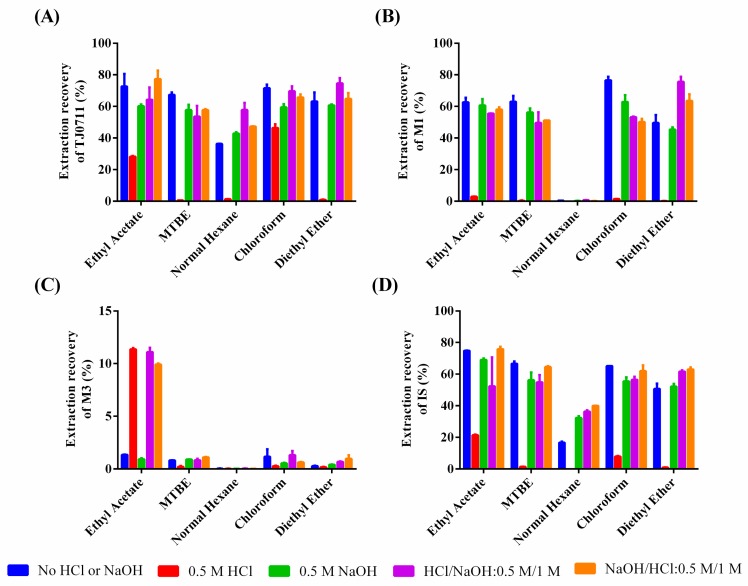
Extraction recovery of TJ0711 (**A**), M1 (**B**), M3 (**C**), and IS (**D**) under various extraction conditions using liquid-liquid extraction. Data are represented as mean ± S.D., *n* = 3.

For sample clean-up, two different types of sample pretreatment methods, liquid-liquid extraction (LLE), and protein precipitation (PPT) were evaluated. In our study, five extraction solvents including ethyl acetate, hexane, chloroform, methyltertiarybutyl ether (MTBE), and diethyl ether were tested. Due to alkalescence of the analytes and IS, 20 µL of 0.5 M sodium hydroxide was used to enhance the extraction recovery for both analytes and IS. However, M3 has a carboxylate radical and, thus, existed as an ionic type under an alkaline condition. Therefore, the extraction process was optimized by adding sodium hydroxide and/or hydrochloric acid (Figure 2). Relatively high extraction recovery of TJ0711, M1, and IS were observed with 0.5 M sodium hydroxide added before extraction. Unfortunately, such acidification and alkalization strategies had little effect on improving the extraction recovery of M3. It is worth noting that a dramatic difference of recovery between the analyte and IS might result in severe precision/accuracy problems. As a result, PPT was developed in this study to achieve high extraction recovery of all analytes of interest. To enhance the MS response, the supernatant after PPT was evaporated using an SPD1010 SpeedVac Concentrator and then reconstituted in 80 μL of 15% ACN in water.

### 3.2. Method Validation

#### 3.2.1. Selectivity and Specificity

**Figure 3:**
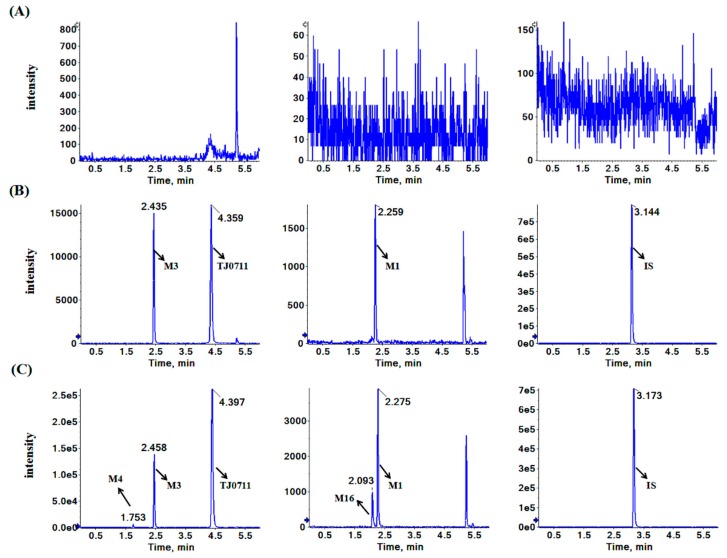
Representative multiple reaction monitoring chromatograms of TJ0711, M1, M3, and IS in dog plasma: (**A**) double blank dog plasma, (**B**) a plasma sample of LLOQ containing TJ0711 M1, M3, and IS, and (**C**) a plasma sample collected at 120 min after intravenous infusion of 3 μg/kg/min TJ0711 in dog (The concentrations were calculated to be 62.5, 0.835, and 28.6 ng/mL for TJ0711, M1, and M3, respectively).

Double blank samples from six individual dogs were compared with the LLOQ sample. The typical chromatograms of double blank sample, the LLOQ sample, and a sample collected 120 min after dosing are shown in Figure 3. No notable interference was found in the retention time of TJ0711, M1, M3, and IS. Moreover, M4 and M16 achieved baseline separation from M3 and M1, respectively, which suggests a good specificity of this method. Additionally, there was no cross-talk between TJ0711, its metabolites, and IS.

#### 3.2.2. LOD and LLOQ

The LOD of TJ0711, M1, and M3 were 0.01 ng/mL of which S/N was larger than 3. The LLOQ was 0.5, 0.1, and 0.5 ng/mL for TJ0711, M1, and M3, respectively. The accuracy and precision of LLOQ met acceptance criteria and the signal-to-noise values of each analyte in LLOQ were greater than 10, which indicated that the established method was sensitive for the quantification and pharmacokinetic analysis of TJ0711, M1, and M3 in dog plasma.

#### 3.2.3. Calibration Curve and Linearity

In this method, eight concentration sets were used to construct the calibration plots with a weighting factor 1/x^2^ to assess linearity. The range of linearity for TJ0711 and M3 was 0.5 to 100 ng/mL and for M1 was 0.1 to 20 ng/mL. Eight calibration standards were performed in triplicate on three separated days. In line with the requirement, eight sets of the calibrators were within <15% (<20% for the LLOQ) of the nominal concentrations. The linear range, regression equations, and correlation coefficients are listed in Table 1. Mandel’s test [29] results demonstrated F_calculated_ values of 23.0, 327.3, and 0.98 for TJ0711, M1, and M3, respectively, and tabulated F_0.05_ (1, 5) value was found to be 6.61, which indicates that TJ0711, and M3 calibration standards were fitted better by a quadratic function. For example, typical calibration curves for TJ0711 and M3 were y = −8.25 × 10^−5^x^2^ + 0.056x + 5.87 × 10^−^^4^ and y = −5.47 × 10^−5^x^2^ + 0.0443x + 5.45 × 10^−5^, respectively. These equations had a significantly smaller coefficient (−5 × 10^−5^~−9 × 10^−5^) for the variable term of “x^2^” compared to the coefficient (×10^−2^) for the variable term of “x”. However, in bioanalytical studies, a linear calibration function was commonly used for calibration curves. Accuracy and precision results calculated by linear calibration fitting were not compromised and also met FDA bioanalysis guidance acceptance criteria. Therefore, a linear calibration function rather than a quadratic function was finally chosen. In addition, both of the function y = ax + b and y = ax were used for the calibration curve assessment of M1. The intercepts of individual calibration curve were calculated by Analyst Software 1.6.1. There were no significant differences (p > 0.05) between the intercepts of two equations. Thus, the function y = ax instead of y = ax + b was employed for the corrected calibration equation of M1 (Table 1).

#### 3.2.4. Accuracy and Precision

Table 2 lists results of accuracy and precision for each LLOQ and QC sample via standard deviation (SD), variable coefficient (CV), and accuracy values. The intra-assay accuracy range of TJ0711, M1, and M3 was 96.27% to 104.00%, 104.00% to 112.67%, and 105.33% to 114.00%, respectively. The inter-assay accuracy range was 94.40% to 102.00%, 96.00% to 105.00%, and 96.00% to 105.33%, respectively. The intra-assay precision (RSD) was <2.98%, <5.64%, and <3.62%, while the inter-assay precision (RSD) was <8.69%, 10.04%, and 11.73%, for TJ0711, M1, and M3, respectively. The results suggested that this method was accurate and precise for analysis of TJ0711, M1, and M3.

#### 3.2.5. Matrix Effect and Extraction Recovery

Matrix effect and extraction recovery were investigated at three QC concentrations and the data are listed in Table 3. There was no notable matrix effect (below 15%) at each concentration examined for each analyte, which indicates that the ionization of neither analyte nor IS was influenced by endogenous compounds in dog plasma. Furthermore, the extraction recovery of each concentration level was >80% for TJ0711, >90% for M1, >85% for M3, and >60% for IS, all with CV < 15%. Although the difference of extraction recovery between analytes and IS was found, it did not affect the accuracy and reliability in the quantification of analytes. In addition, there was no significant difference among three concentration levels in extraction recovery. The matrix effects of hemolytic samples were also evaluated and no notable matrix effects were observed (Table S1).

#### 3.2.6. Dilution Integrity

Six replicate plasma samples with a concentration above the ULOQ were diluted 5-fold and 10-fold with blank dog plasma, respectively. The concentrations of 5-fold and 10-fold diluted samples were within ±15% of the nominal concentration in six replicates (data not shown).

#### 3.2.7. Carry-Over

A double blank sample was run after the ULOQ sample to observe carry-over. No peak was found in the double blank sample after the ULOQ sample for both analytes and IS, which verified that there was no carry-over in the method.

#### 3.2.8. Stability

The stability of TJ0711, M1, and M3 in dog plasma was evaluated using LQC and HQC samples and the results are presented in Table 4. TJ0711, M1, M3, and IS were stable in the stock solution for 34 days and for 30 days in working solutions at 4 °C. The analytes in the biological matrices were proven to be stable over three freeze-thaw cycles, after storage at room temperature for 24 h, and after storage at −80 °C for 30 days. Moreover, TJ0711, M1, and M3 in the final extract were stable after storage at room temperature for 24 h and after storage at an auto sampler temperature for 24 h. In addition, the stability of analytes in whole blood was also evaluated. Based on the result, TJ0711, M1, and M3 were stable in whole blood both at room temperature and on wet ice (Table S2). 

### 3.3. Preclinical Pharmacokinetic Study

**Figure 4:**
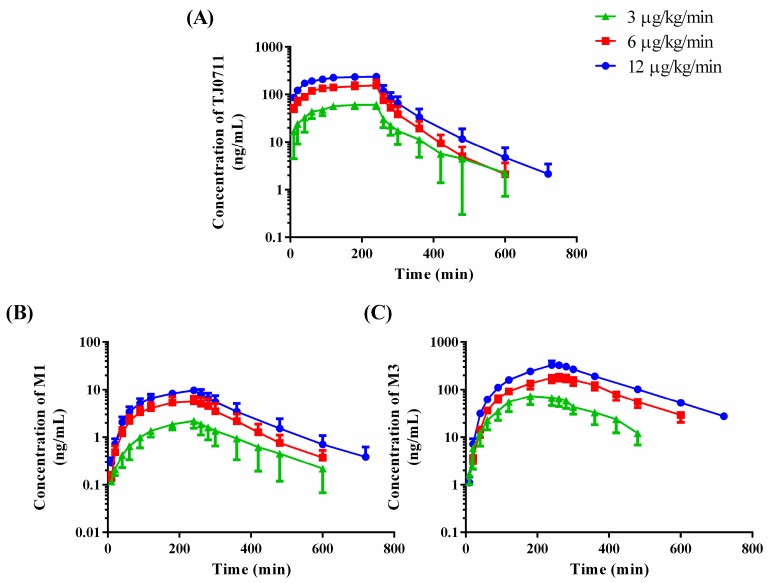
Plasma concentration–time profiles for TJ0711 (**A**), M1 (**B**), and M3 (**C**) in beagle dogs following intravenous infusion of 3, 6, and 12 μg/kg/min TJ0711·HCl. Data are represented as mean ± S.D, *n* = 6.

**Figure 5:**
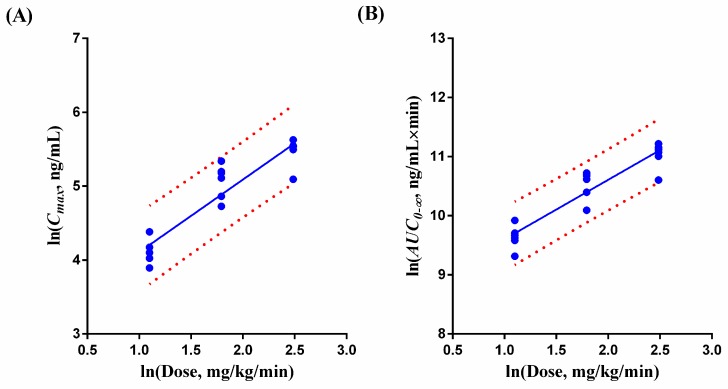
Relationship between ln(PK) and ln(Dose). (**A**) *C_max_*, (**B**) *AUC*_0–∞_. The full lines are the fitted values calculated by the power model and the dotted lines are the 95% prediction band.

According to the previous pharmacokinetic/pharmacodynamic studies after a single intravenous injection of TJ0711 in conscious beagle dogs (data not published), the blood pressure decreased by 20% when plasma TJ0711 concentration reached 100 ng/mL, which was recognized as an effective antihypertensive concentration. Based on clearance and half life results of TJ0711, 6 µg/kg/min was selected as a medium dosing rate of our study. Then, 3 µg/kg/min and 12 µg/kg/min were chosen to be low and high dosing rate. The mean measured plasma concentration versus time profiles of TJ0711, M1, and M3 are presented in Figure 4. Pharmacokinetic parameters are listed in Table 5. TJ0711 was mainly bio-transformed into M3 after intravenous infusion in the beagle dog. However, the *AUC*_0–*t*_ of M1 did not exceed 5% of TJ0711, which might relate to the weak metabolic stability of M1 in dog liver microsomes. In the in vitro metabolic stability of M1, the remaining percentage of M1 was above 70% after 60 min incubation in 0.25 mg/mL human liver microsomes, while the elimination of M1 was faster in dog liver microsomes, which indicates significant differences between species in metabolic stability of M1 [14]. A power function model was applied to evaluate the proportionality between dose and PK parameters (Figure 5A for *C_max_* of TJ0711 and Figure 5B for *AUC*_0–∞_ of TJ0711). The proportionality constant β (95% CI) for *C_max_* and *AUC*_0–∞_ of TJ0711 were 0.9819 (0.7745–1.1890) and 1.0050 (0.7958–1.2140), respectively, which indicates linear does-proportionality. Moreover, M1 and M3 also exhibited good does-proportionality in the dosage of 3–12 µg/kg/min (data shown in Figure S1). In addition, no significant differences were observed either for *t*_1/2_, *CL*, or *V_ss_* values of TJ0711 or for *t*_1/2_ values of metabolites among three dose groups. The results above suggested that the disposition processes of TJ0711 and its metabolite in beagle dogs followed linear pharmacokinetics. It was notable that half-lives and *V_d_* in the current study were greater (about 1.75-fold and 2-fold, respectively) compared to our previous study [15]. In the present PK study, isoflurane, which is a kind of inhalational anesthetics, was used to anesthetize beagle dogs. It has been reported that inhalational anesthetics might affect the distribution, hepatic blood flow, or metabolism of perioperative drugs [26]. The difference between these pharmacokinetic parameters might be due to the differences in drug disposition under anesthesia and consciousness state. It was worth noting that the different pharmacokinetic profiles between conscious and anesthetic states might occur in clinic used of TJ0711 in future. Cautions must be taken when administrated TJ0711 under anesthetic states. Anti-hypertensive effects can be controlled by adjusting the rate of administration if necessary. Lastly, 60 incurred samples were re-analyzed and all results met the acceptance criteria, which confirmed the reproducibility of the proposed method.

## 4. Conclusions

In our study, a rapid, selective, and reliable LC-MS/MS method was developed and validated for the simultaneous quantitation of TJ0711 and two metabolites (M1 and M3) in beagle dog plasma. Due to the low extraction recovery of M3 in five extraction agents under various extraction conditions of LLE, PPT followed by evaporation and reconstitution was used to prepare plasma samples, which leads to a high MS response and no matrix effect. The validated method was successfully applied to pharmacokinetic study in beagle dogs following intravenous infusion of TJ0711∙HCl. The PK profiles of TJ0711 and its two metabolites followed linear process between the dosing rates of 3 to 12 µg/kg/min. Furthermore, the differences of the PK process were found between conscious and anesthetized dogs, which indicates that caution should be taken in clinical use of TJ0711 under conscious and anesthetic states.

## Figures and Tables

**Table 1:** Linear range, accuracy, regression equations, and correlation coefficients.

Analyte	Nominal Conc. (ng/mL)	Accuracy (%)	Analyte	Nominal Conc. (ng/mL)	Accuracy (%)	Analyte	Nominal Conc. (ng/mL)	Accuracy (%)
TJ0711	0.5	98.80	M1	0.1	102.00	M3	0.5	99.80
	1	96.90		0.2	103.00		1	96.50
	2	107.00		0.5	100.00		2	104.00
	5	109.00		1	110.00		5	109.20
	20	101.50		5	98.60		20	102.00
	50	100.40		10	98.30		50	100.20
	90	96.44		18	96.67		90	97.56
	100	89.50		20	92.00		100	90.70
Regression equation	y = 0.0519x + 0.0034			y = 0.0185x			y = 0.0415x + 0.00192	
Correlation coefficient (r)	0.9975			0.9989			0.9980	

**Table 2:** Intra-assay and inter-assay accuracy and precision summary of analytes in dog plasma.

Analyte	Nominal Conc. (ng/mL)	Intra-Assay (*n* = 6)				Inter-Assay (*n* = 18)			
		Measured Conc. (ng/mL)		Accuracy (%)	Precision (CV, %)	Measured Conc. (ng/mL)		Accuracy (%)	Precision (CV, %)
		Mean	SD			Mean	SD		
TJ0711	0.5	0.515	0.015	103.00	2.98	0.491	0.043	98.20	8.69
	1.5	1.55	0.05	103.33	2.90	1.53	0.04	102.00	2.43
	10	10.4	0.2	104.00	1.49	10.1	0.3	101.00	2.95
	75	72.2	0.9	96.27	1.27	70.8	3.5	94.40	4.91
M1	0.1	0.112	0.006	112.00	4.93	0.107	0.010	107.36	9.36
	0.3	0.318	0.010	106.00	3.22	0.309	0.031	103.04	10.04
	2	2.09	0.07	104.33	3.42	2.02	0.18	100.75	8.79
	15	14.6	0.3	97.22	1.96	14.4	1.1	95.70	7.58
M3	0.5	0.547	0.020	109.40	3.62	0.499	0.059	99.80	11.73
	1.5	1.71	0.05	114.00	3.12	1.58	0.12	105.33	7.79
	10	11.3	0.2	113.00	2.14	10.5	0.8	105.00	7.77
	75	79.1	1.5	105.33	1.86	72.0	6.3	96.00	8.70

**Table 3:** The matrix effect and extraction recovery of TJ0711, M1, M3, and IS in dog plasma (*n* = 6).

Analyte	Nominal Conc. (ng/mL)	Matrix Effect (%)			Extraction Recovery (%)		
		Mean (%)	SD (%)	CV (%)	Mean (%)	SD (%)	CV (%)
TJ0711	1.5	93.31	1.25	1.34	85.60	1.25	1.46
	10	95.80	3.50	3.65	84.75	1.13	1.33
	75	94.56	1.48	1.56	82.82	2.32	2.80
M1	0.3	103.21	4.41	4.28	97.82	2.88	2.95
	2	104.04	5.33	5.12	97.63	5.41	5.54
	15	100.18	3.64	3.63	92.25	2.69	2.92
M3	1.5	93.12	2.18	2.34	94.09	2.38	2.53
	10	95.10	4.74	4.99	91.97	1.08	1.17
	75	93.29	1.84	1.97	89.84	1.83	2.04
IS	100	90.11	2.42	2.69	62.05	0.70	1.12

**Table 4:** Stability of TJ0711, M1, and M3 in dog plasma under various conditions (*n* = 6).

Analyte	Nominal Conc. (ng/mL)	Storage Condition	Accuracy (%)	CV (%)
TJ0711	1.5	Auto sampler (4 °C, 24 h)	100.44	2.17
		BT ambient temperature (24 h)	99.11	1.84
		Plasma FT-3 cycles	104.44	2.92
		Plasma 30 days at −80 °C	99.22	2.23
	75	Auto sampler (4 °C 24 h)	91.47	2.52
		BT ambient temperature (24 h)	98.76	14.18
		Plasma FT-3 cycles	92.96	2.57
		Plasma 30 days at −80 °C	90.20	3.42
M1	0.3	Auto sampler (4 °C, 24 h)	103.00	4.82
		BT ambient temperature (24 h)	101.11	3.42
		Plasma FT-3 cycles	98.33	4.87
		Plasma 30 days at −80 °C	100.17	3.63
	15	Auto sampler (4 °C, 24 h)	88.78	2.25
		BT ambient temperature (24 h)	88.44	2.27
		Plasma FT-3 cycles	90.33	3.52
		Plasma 30 days at −80 °C	88.22	3.75
M3	1.5	Auto sampler (4 °C, 24 h)	102.33	3.10
		BT ambient temperature (24 h)	101.11	2.94
		Plasma FT-3 cycles	103.78	3.29
		Plasma 30 days at −80 °C	98.78	2.43
	75	Auto sampler (4 °C, 24 h)	90.47	3.16
		BT ambient temperature (24 h)	88.33	3.10
		Plasma FT-3 cycles	91.64	3.71
		Plasma 30 days at −80 °C	87.51	4.12

BT: Bench-top.

**Table 5:** The pharmacokinetic parameters of TJ0711 after intravenous infusion for 4 h in beagle dogs (*n* = 6).

Analyte	Parameters	Unit	Dose (μg/kg/min)		
			3	6	12
TJ0711	*C_max_*	ng/mL	61.85 ± 10.39	162.58 ± 35.31	241.92 ± 40.23
	*T_max_*	min	200.0 ± 49.0	220.0 ± 31.0	240.0 ± 0.0
	*AUC*_0–*t*_	ng/mL × min	15,277.2 ± 2799.7	38,202.2 ± 8225.1	62,255.6 ± 11,680.1
	*AUC*_0–∞_	ng/mL × min	15,561.2 ± 3004.5	38,442.9 ± 8306.7	62,827.7 ± 12,015.0
	*t*_1/2_	min	70.1 ± 16.1	68.2 ± 14.5	78.8 ± 11.1
	*CL*	mL/min	494.61 ± 94.46	426.83 ± 119.14	512.27 ± 139.11
	*V_ss_*	L	35.5 ± 15.9	24.8 ± 2.8	27.4 ± 2.9
	*V_d_*	L	49.2 ± 10.0	40.8 ± 8.6	57.3 ± 12.4
M1	*C_max_*	ng/mL	2.23 ± 0.66	5.93 ± 1.79	9.86 ± 1.36
	*T_max_*	min	230.0 ± 24.5	230.0 ± 24.5	243.0 ± 8.2
	*AUC*_0–*t*_	ng/mL × min	591.7 ± 215.6	1550.2 ± 517.5	2604.7 ± 699.9
	*AUC*_0–∞_	ng/mL × min	626.1 ± 239.0	1602.0 ± 532.2	2669.9 ± 741.5
	*t*_1/2_	min	103.9 ± 10.9	98.2 ± 18.0	109.1 ± 20.4
M3	*C_max_*	ng/mL	73.23 ± 24.40	188.75 ± 36.50	339.83 ± 63.26
	*T_max_*	min	250.0 ± 24.5	256.7 ± 24.5	246.7 ± 17.4
	*AUC*_0–*t*_	ng/mL × min	21,502.2 ± 6670.4	55,593.8 ± 10712.7	101,567.3 ± 11,051.9
	*AUC*_0–∞_	ng/mL × min	23,837.4 ± 7059.2	61,441.5 ± 10,939.6	106,811.2 ± 10,493.2
	*t*_1/2_	min	130.9 ± 36.9	133.6 ± 34.0	129.7 ± 17.4

## Supplementary Materials

The following are available online at http://www.mdpi.com/1999-4923/11/1/38/s1, Figure S1. Relationship between ln(PK of metabolites) and ln(Dose of TJ0711). (A) *C_max_* of M1. (B) *AUC*_0–∞_ of M1. (C) *C_max_* of M3. (D) *AUC*_0–∞_ of M3. The full lines are the fitted values calculated by the power model and the dotted lines, which are the 95% prediction band. Table S1. Matrix effect of TJ0711, M1, M3, and IS in hemolysis sample (*n* = 6). Table S2. The stability of analytes in whole blood (*n* = 3).
