# Supplementary material for: Simultaneous Quantitation of a Novel α1/β1-Blocker TJ0711 and Its Two Metabolites in Dog Plasma Using LC-MS/MS and Its Application to a Pharmacokinetic Study after Intravenous Infusion
Source: Pharmaceutics. 2019 Jan 18;11(1):38. doi: 10.3390/pharmaceutics11010038 (PMC6359546; doi:10.3390/pharmaceutics11010038)
Supplement: Supplementary file 1 [file pharmaceutics-11-00038-s001.pdf]

pharmaceutics

Supplementarty Materials: Simultaneous Quantitation of a Novel α_1/β_1-Blocker TJ0711 and Its Two Metabolites in Dog Plasma Using LC-MS/MS and Its Application to a Pharmacokinetic Study after Intravenous Infusion

Wenwen Zhu, Wanyu Liu, Haojv Li, Guojia Xu, Qian Li, Jiangeng Huang, Gao Li and Luqin Si

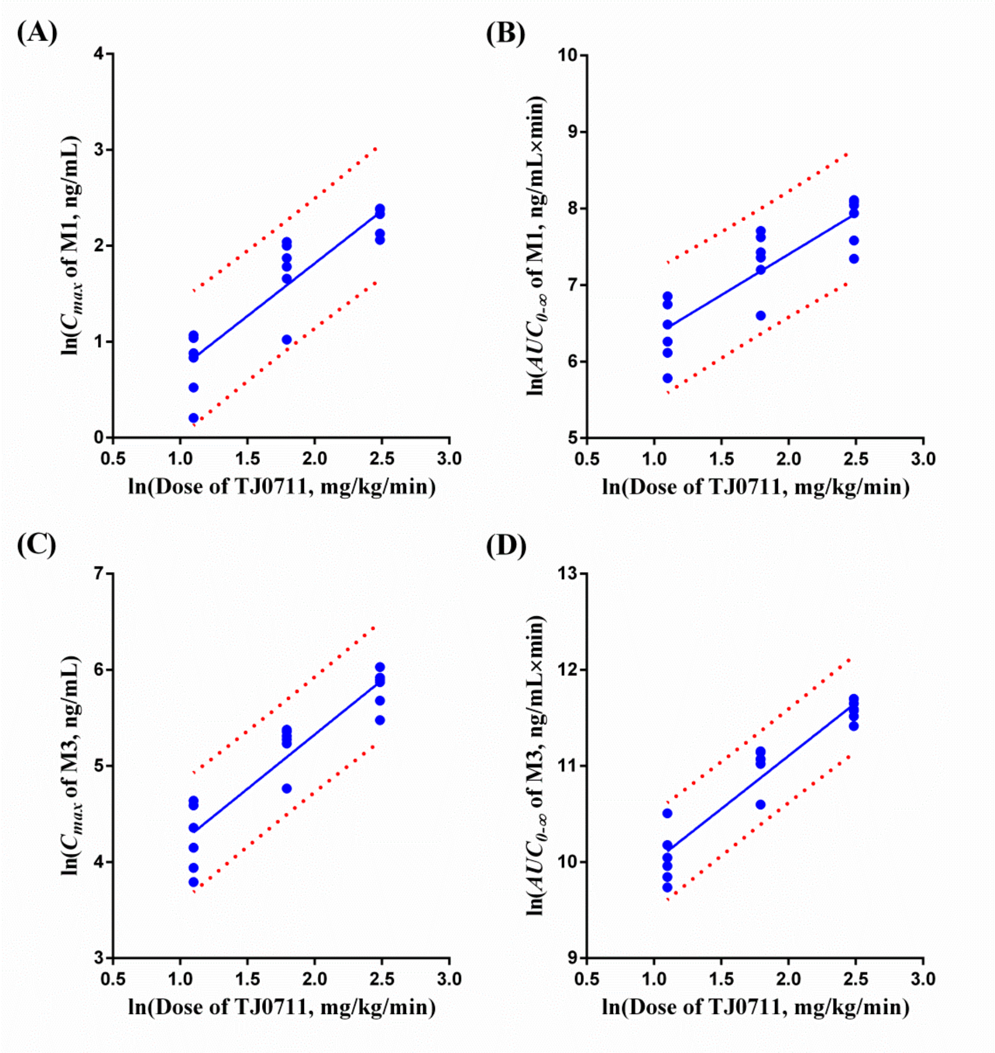

Figure 1. Relationship between ln(PK of metabolites) and ln(Dose of TJ0711). (**A**) C_{max} of M1. (**B**) $AUC_{0-\infty}$ of M1. (**C**) C_{max} of M3. (**D**) $AUC_{0-\infty}$ of M3. The full lines are the fitted values calculated by the power model and the dotted lines, which are the 95% prediction band.

Table S1. Matrix effect of TJ0711, M1, M3, and IS in hemolysis sample (n = 6).

Analyte	Nominal Conc. (ng/mL)	Matrix effect (%)		
		Mean (%)	SD (%)	CV (%)
TJ0711	1.5	92.99	2.49	2.68

	10	95.86	1.32	1.38
	75	100.50	2.41	2.39
M1	0.3	102.46	1.86	1.91
	2	96.05	3.34	3.47
	15	100.29	3.90	3.89
M3	1.5	93.05	3.65	3.92
	10	94.12	2.40	2.55
	75	97.45	2.60	2.67
IS	100	91.58	2.82	3.07

Table S2. The stability of analytes in whole blood (*n* = 3).

Analyte	Conditions	Spiked Conc.	Area ratio (mean ± SD)	Ratio difference
TJ0711	Centrifuged immediately	1.5	0.1058 ± 0.0110	-
		75	4.0215 ± 0.0588	-
	BT ambient temperature (2 h)	1.5	0.1097 ± 0.0026	3.55
		75	4.3396 ± 0.1172	7.61
	Wet ice (2 h)	1.5	0.0937 ± 0.0022	−12.16
		75	3.9206 ± 0.0287	−2.54
M1	Centrifuged immediately	0.3	0.0937 ± 0.0022	-
		15	3.9206 ± 0.0287	-
	BT ambient temperature (2 h)	0.3	0.0050 ± 0.0002	5.83
		15	0.2348 ± 0.0027	3.53
	Wet ice (2 h)	0.3	0.0053 ± 0.0003	−3.97
		15	0.2433 ± 0.0052	−3.29
M3	Centrifuged immediately	1.5	0.0048 ± 0.0003	-
		75	0.2272 ± 0.0035	-
	BT ambient temperature (2 h)	1.5	0.1058 ± 0.0110	3.55
		75	4.0215 ± 0.0588	7.61
	Wet ice (2 h)	1.5	0.1097 ± 0.0026	−12.16
		75	4.2296 ± 0.1172	−2.54

*BT: Bench-top.
